# Altered Expression of the Malate-Permeable Anion Channel OsALMT4 Reduces the Growth of Rice Under Low Radiance

**DOI:** 10.3389/fpls.2018.00542

**Published:** 2018-05-03

**Authors:** Jie Liu, Muyun Xu, Gonzalo M. Estavillo, Emmanuel Delhaize, Rosemary G. White, Meixue Zhou, Peter R. Ryan

**Affiliations:** ^1^Agriculture and Food, Commonwealth Scientific and Industrial Research Organisation, Canberra, ACT, Australia; ^2^College of Agronomy, Sichuan Agricultural University, Chengdu, China; ^3^School of Land and Food and Tasmanian Institute for Agriculture, University of Tasmania, Hobart, TAS, Australia

**Keywords:** malate, rice, roots, photosynthesis, stress, channel, OsALMT4

## Abstract

We examined the function of *OsALMT4* in rice (*Oryza sativa* L.) which is a member of the aluminum-activated malate transporter family. Previous studies showed that OsALMT4 localizes to the plasma membrane and that expression in transgenic rice lines results in a constitutive release of malate from the roots. Here, we show that *OsALMT4* is expressed widely in roots, shoots, flowers, and grain but not guard cells. Expression was also affected by ionic and osmotic stress, light and to the hormones ABA, IAA, and salicylic acid. Malate efflux from the transgenic plants over-expressing *OsALMT4* was inhibited by niflumate and salicylic acid. Growth of transgenic lines with either increased *OsALMT4* expression or reduced expression was measured in different environments. Light intensity caused significant differences in growth between the transgenic lines and controls. When day-time light was reduced from 700 to 300 μmol m^-2^s^-1^ independent transgenic lines with either increased or decreased *OsALMT4* expression accumulated less biomass compared to their null controls. This response was not associated with differences in photosynthetic capacity, stomatal conductance or sugar concentrations in tissues. We propose that by disrupting malate fluxes across the plasma membrane carbon partitioning and perhaps signaling are affected which compromises growth under low light. We conclude that OsALMT4 is expressed widely in rice and facilitates malate efflux from different cell types. Altering *OsALMT4* expression compromises growth in low-light environments.

## Introduction

Aluminum-activated malate transporters (ALMT) form a family of anion channels in plants (Sasaki et al., 2004; Punta et al., 2012; Sharma et al., 2016). Anion channels are membrane-bound proteins that facilitate the energetically passive transport of anions across membranes. Plant cells generate a negative electrical potential difference across the plasma membrane and the tonoplast such that anion channels generally mediate anion movement from the cytosol to the apoplast or into the vacuole. Anion channels contribute in many basic cellular functions including signaling, charge balancing and controlling membrane potential, substrate compartmentation, osmotic control, turgor regulation and responses to nutrient deficiency and abiotic stress (Barbier-Brygoo et al., 2011).

*ALMTs* are widely distributed in the genomes of higher plants as well as the Bryophyta with 14 members in *Arabidopsis thaliana* and nine in rice (*Oryza sativa* L.) (Delhaize et al., 2007; Barbier-Brygoo et al., 2011; Dreyer et al., 2012; De Angeli et al., 2013b). ALMT proteins typically have five to seven transmembrane regions in the N-terminal half and a long hydrophilic C-terminal tail but predictions of secondary structure vary (Motoda et al., 2007; Dreyer et al., 2012).

Some members of the family function as aluminum (Al^3+^)-activated anion channels which protect the roots from aluminum toxicity by facilitating malate efflux from the root apices. These have been reported in Sasaki et al. (2004), Arabidopsis (Hoekenga et al., 2006), *Brassica napus* (Ligaba et al., 2006), rye (*Secale cereale* L.) (Collins et al., 2008), soybean (*Glycine max* L.) (Liang et al., 2013), *Medicago sativa* (Liang et al., 2013), and *Holcus lanatus* (Chen et al., 2013). Most members of the ALMT family are not involved in Al^3+^ resistance but localize to various membranes and perform other functions (Barbier-Brygoo et al., 2011; Sharma et al., 2016). For instance, at least four members in Arabidopsis (AtALMT4, AtALMT6, AtALMT9, and AtALMT12) and one in barley (*Hordeum vulgare*; HvALMT1) are expressed in guard cells which is consistent with the involvement of anion transport in signal transduction and osmotic adjustment to control stomatal aperture (Gruber et al., 2010; Meyer et al., 2010b; Sasaki et al., 2010; Dreyer et al., 2012; De Angeli et al., 2013a; Xu et al., 2015). AtALMT4, AtALMT6, and AtALMT9 localize to the tonoplast and AtALMT12 localizes to the plasma membrane. AtALMT4 is required for stomatal closure because it facilitates malate transport out of the vacuole during abscisic acid-induced stomatal closure, when the tonoplast membrane potential is less negative (Eisenach et al., 2017). OsALMT9 is expressed widely in Arabidopsis (Kovermann et al., 2007) and is important for the regulation of stomatal opening because it facilitates the malate-induced transport of Cl^-^ into the vacuole (De Angeli et al., 2013a). AtALMT6 facilitates malate transport into the vacuole but *almt6* knock-out mutants show no changes to guard cell function (Meyer et al., 2011). When expressed in *Xenopus* oocytes AtALMT12 generates similar currents to the previously characterized rapid or R-type/QUAC anion currents (Hedrich, 2012). This prompted Meyer et al. (2010b) to propose that AtALMT12 anion channels are responsible for these currents. Other members in grape (*Vitis vinifera* L.) and apple (*Malus domestica*) reportedly regulate the transport of malate and other organic anions into the vacuole of fruit tissues (Bai et al., 2012; De Angeli et al., 2013a). In maize, *ZmALMT1* and *ZmALMT2* are mainly expressed in root tissues and likely function in balancing charges during nutrient uptake (Pineros et al., 2008; Ligaba et al., 2012).

A phylogenetic tree of the ALMT family clusters its members into five clades. Clade 5 is unusual because it includes two closely related members from rice, OsALMT4 and OsALMT5, but none from Arabidopsis genome (Dreyer et al., 2012; Liu et al., 2017). We previously investigated these two genes and found that *OsALMT5* contained a mutation that would lead to a truncated protein. Our initial characterization of OsALMT4 showed that it localizes to the plasma membrane and facilitates the release of malate anions from cells (Liu et al., 2017). The present study examined the biology of *OsALMT4* in more detail by examining where it is expressed in rice and how expression is affected by a range of treatments. To examine protein function we also investigated how the malate efflux from transgenic plants is modulated by various treatments and how altering *OsALMT4* expression affected growth in different environments.

## Materials and Methods

### Plant Material and Growth Conditions

Rice (*Oryza sativa* L.; cv *Nipponbare*) seed were obtained from the collection at CSIRO. Seed were surface sterilized by 20% bleach for 15 min, rinsed thoroughly in water and pre-germinated under dark on ½ MS media for 2 days at 28°C. Germinated seedlings were then grown under continuous light for 5 days and moved to soil or to hydroponics in either tanks (10 L) or flasks. Hydroponic solution consisted of ½ strength Kimura B solution where the final concentrations were 250 μM KNO_3_, 250 μM CaCl_2_, 250 μM NH_4_NO_3_, 75 μM MgSO_4_, 10 μM KH_2_PO_4_, 0.4 μM EDTA:Fe, 5.5 μM H_3_BO_3_, 1 μM MnCl_2_, 0.175 μM ZnCl_2_, and 0.1 μM CuCl_2_, adjusted to pH 5.6. Solution were changed regularly. Growth in flooded conditions used pots (8.5 cm diameter and 20 cm high) and “rice soil” prepared at CSIRO which were maintained in glasshouses or growth cabinets.

### Tissue Specific Expression

The 2,496 bp region upstream of the *OsALMT4* transcription start site was amplified from genomic DNA using primers (5′–3′) CCTTAATTAAACCTGTTACTACTTGTTATGC (forward) and TTGGCGCGCCTCTCTAACTTGCGGTCTCTT (reverse) which introduced *Pac*I and *Asc*I sites at each end. This was used to drive GFP expression. The amplicon was cloned and ligated into the plasmid “PS1aGFP-OsALMT4_promoter” (Gruber et al., 2010). The plasmid was transformed into *E. coli* DH5α and positive clones were detected by PCR and enzyme digestion. Positive clones were then transformed into competent cells of AGL-1 agrobacterium and prepared for rice transformation. Once the primary transgenic plants (T_0_) developed roots they were transferred to hydroponics so different tissues could be examined 2 weeks later under a fluorescent microscope. Plants were then transferred to soil to monitor GFP expression in the flowers and developing grain of larger plants. The flower tissues were collected at ∼10:00 am on the day of flowering. T_1_ seed collected from the T_0_ plants was germinated and seedlings examined at different stages of development. Developing grains were collected and checked at 14 days (for milk stage) and 28 days (for dough stage) after flowering and the germination of mature grain was monitored daily for 10 days after germination. GFP fluorescence was examined and photographed with a Leica MZFLIII fluorescence dissecting microscope (Excitation filter: 425–60 nm, Barrier filter: 480 nm) and Leica SP2 confocal laser scanning microscope (excitation wavelength was 488 nm and emission collection bin was 480–600 nm). Plant tissues were examined as quickly as possible after dissection to eliminate the autofluorescence which is sometimes associated with cell death. Untransformed wild-type (WT) rice plants were grown in parallel to control for the autofluorescence that occurred in some tissues. The results presented here all showed distinct differences in fluorescence between WT and transgenic plants.

### Expression of *OsALMT4* in Wild-Type Plants During Different Treatments

Seed were germinated as described above and transferred to hydroponics (pH 5.6) with a diurnal cycle. After 7 days the solutions were changed and different treatments applied for either 6 or 24 h. Osmotic treatments included 200 mM NaCl, 350 mM mannitol (which have approximately the same osmotic pressure) and 20% PEG6000. Other treatments included sustained high light (∼500 μmol m^-2^s^-1^) or sustained darkness, an acidic solution (pH 4.5) with and without 100 μM AlCl_3_, a low Cl^-^ solution (2.6 μM) and 300 μM hydrogen peroxide to trigger oxidative stress. The low Cl^-^ treatment was prepared by replacing CaCl_2_ with CaSO_4_ in the standard hydroponic solution. Hormone treatments (100 μM) were abscisic acid (ABA), indole-3-acetic acid (IAA), salicylic acid (SA), gibberellin (GA_3_), methyl jasmonic acid (MeJA) and finally γ-amino butyric acid (GABA; 1 mM). After treatment, the roots and first fully expanded leaf were collected for expression analysis. Quantitative RT-PCR was performed with *OsALMT4*-specific primers CCTTAGAAGAGTGTGTCAAGAAG (forward) and CCATTTAGCAGAGTTCGCCAG (reverse) using SYBR Green (Bio-Rad) and the CFX96 Real-Time System (Bio-Rad) following the manufacturer’s instructions. Reactions were performed with three biological replicates from three technical replicates for each sample. Transcript levels of *OsALMT4* were normalized with two reference genes: *glyceraldehyde-3-phosphate dehydrogenase* (*GAPDH*, GenBank: GQ848049.1) using primers GTTGAGGGTTTGATGACCAC (forward) and CAGACTCCTCCTTGATAGC (reverse) and *elongation factor-1 alpha* (*eEF-1a*, GenBank: GQ848073.1) using primers TTTCACTCTTGGTGTGAAGCAGAT (forward) and GACTTCCTTCACGATTTCATCGTAA (reverse) to provide a single result according to the software in Bio-Rad CFX Manager^TM^.

### Metabolomics Analyses

Samples were extracted and analyzed by Metabolomics Australia at The University of Melbourne, a NCRIS initiative under Bioplatforms Australia Pty Ltd as described by Dias et al. (2015). Briefly, transgenic rice plants were germinated and grown in hydroponics in a growth cabinet (28/22°C, 16 h/8 h day/night, light intensity 700 μmol m^-2^ s^-1^) for 35 days. Leaf and root tissues were harvested, immediately frozen in liquid nitrogen and freeze-dried for 72 h. Aliquots (∼20 mg) of freeze-dried root and shoot material were transferred to Cryo-mill tubes and accurate weights recorded. Aliquots of 600 μL of methanol (MeOH) containing internal standards, ^13^C_6_-Sorbitol (0.01 mg.mL^-1^) and ^13^C_5_-^15^N-Valine (0.01 mg.mL^-1^) were added to the sample tubes. Samples were then homogenized using a Cryo-mill (Bertin Technologies; program #2; 6800-3x30x30 at -10°C) followed by 15 min incubation in a Thermomixer at 30°C at 1,400 rpm. Samples were then centrifuged for 15 min at ambient temperature at 13,000 rpm (15,900 × *g*). Supernatants were then transferred into fresh 2 mL Eppendorf tubes. Aliquots of 600 μL water (H_2_O) were added to the remaining pellet and vortexed for 1 min. Samples were then centrifuged for 15 min at ambient temperature at 13,000 rpm (15,900 × *g*). The resulting supernatant was then removed and combined with the first supernatant. For GCMS quantification of sugars, aliquots of 10 μL and 100 μL of sample extracts were transferred to clean glass inserts in Eppendorf tubes and dried *in vacuo* using a Rotational Vacuum Concentrator (RVC 2-33 CD plus, John Morris Scientific, Pty Ltd., Melbourne, VIC, Australia) set at ambient temperature. The dried aliquots were re-dissolved in 20 μL of 30 mg.mL^-1^ methoxyamine hydrochloride in pyridine and derivatized at 37°C for 120 min with mixing at 500 rpm. Samples were then treated for 30 min with 20 μL *N*,*O*-bis-(trimethylsilyl)trifluoroacetamide with 1% trimethylchlorosilane (BSTFA + 1% TMCS). Each derivatized sample was allowed to rest for 60 min prior to injection. Samples (1 μL) were injected into a GC-QqQ-MS system comprised of a Gerstel 2.5.2 Autosampler, a 7890A Agilent gas chromatograph and a 7000 Agilent triple-quadrupole MS (Agilent, Santa Clara, CA, United States) with an electron impact (EI) ion source. The GC was operated in constant flow mode with helium as the carrier gas at a flow rate of 1 mg.mL^-1^. The MS was adjusted according to the manufacturer’s recommendations using tris-(perfluorobutyl)-amine (CF43). GC-QqQ-MS data were processed using the Agilent MassHunter Quantitative Analysis version B.07.00 software. Mass spectra of eluting TMS compounds were identified and quantified using corresponding calibration series of authentic standards. Final results were normalized to internal standard, ^13^C_6_-Sorbitol and tissue weight, and expressed as picomoles/mg dried weight.

### Measuring Organic Anion Efflux From Rice Plants

Organic anion efflux was measured from intact seedlings. Seed were surface sterilized, germinated for 2 days and transferred to 125 mL flasks (three per flask) containing 30 ml sterile nutrient solution. The flasks were placed on a shaker (∼70 rpm) at 28°C and low light (∼50 μmol m^-2^s^-1^). After 4 days the solutions were gently poured out from the flasks and seedlings were rinsed twice with the same sterile control solution or a treatment solution. After rinsing, 10 mL of control or treatment solution was added to each flask and returned to the shaker in the same conditions for 6 h or 20 h depending on the experiment. At the end of the incubation period 0.6 mL solution was collected from each flask for malate assays described previously (Ryan et al., 1995). Contaminated flasks were discarded. None of the treatments added to alter malate efflux caused permanent damage to the seedlings because all kept growing once the treatments were replaced with control solution at the end of the experiments.

### Longer Term Growth Experiments

Six-day-old seedlings were grown in standard hydroponics for 2 days in the glasshouse. The solution was then renewed with or without the following treatments: 100 mM NaCl, 50 mM mannitol or a low-Cl^-^ solution (2.6 μM). The 100 mM NaCl solution also included 12 mM CaCl_2_ to minimize the displacement of Ca and subsequent membrane damage. The low Cl^-^ treatment was prepared by replacing CaCl_2_ with CaSO_4_ in the standard hydroponic solution. All the solutions were renewed every 3–4 days and the plants were harvested after 4 weeks. Root and shoot tissues were harvest separately and dried in 70°C oven for 48 h. Root and shoot biomass was combined for total dry weight. Since growth of the transgenic and null plants sometimes differed in control solution they were compared on the basis of relative biomass. This is defined as the ratio (biomass in the treatment/biomass in control). Experiments with the OX lines were conducted at different times from the experiments with the RNAi lines.

For measuring growth at different light intensities seedlings were transferred to pots with soil and placed on trays in growth cabinets (28/22°C, 16 h/8 h day/night). Maximum diurnal light intensities of the cabinets was either 700 μmols m^-2^s^-1^ (control) or 300 μmol m^-2^s^-1^ (low). Shoots were harvested and dried after 56 days for the OX lines and 35 days for the RNAi lines.

### Gas Exchange Measurements

The responses of carbon dioxide assimilation rates (*A*) to varying irradiance were measured on fully expanded flag leaves with a LICOR LI-6400 (Li-Cor, Lincoln, NE, United States). Plants were grown 6 weeks under flooded soil condition in a Phytotron glasshouse with natural lighting and a 32°C/24°C temperature range. Photosynthetic parameters were measured in a 6 cm^2^ chamber, at a target leaf temperature of 30°C, and flow rate of 500 μmol s^-1^. The chamber relative humidity and flow were adjusted to maintain vapor pressure deficit of the leaf between 1.0 and 1.5 kPa. Light response of *A* (light curve) was carried out at a CO_2_ concentration of 400 μmol CO_2_ mol^-1^ (ppm) in the reference cell while varying the light intensities as follows: 2,000, 1,500, 1,000, 500, 250, 60, 0 μmol m^-2^s^-1^. Gas exchange measurement were performed between 9:00 and 13:30 and concurrent SPAD values were scored for each leaf. The theoretical maximum electron transport rate at saturating light (*J*_max_) was estimated from the light curves using the online tool by Sharkey et al. (2007) based on the model of photosynthesis described previously (Farquhar et al., 1980).

### Statistical Analysis

Treatment effects on malate efflux were compared with the Student *t*-test in the SigmaPlot^TM^ version 13 (Systat Software Inc.). Statistical comparison of relative biomass data from the growth experiments first required the standard errors for relative biomass (RB) to be calculated. Standard errors of RB (SE_RB_) were calculated as follows: SE_RB_ = RB × [(SE*x*/*x*)^2^+ (SE*y*/*y*)^2^]^1/2^, where *x* is the mean biomass of the control and SE*x* is the standard error of that mean, and *y* is the mean of the treatment and SE*y* represents the standard error of that mean. Statistical comparisons of the relative biomass of one line with another followed an approach based on overlapping confidence limits (CL) described previously (Zhou et al., 2013).

## Results

### *OsALMT4* Show a Wide Tissue-Specific Expression

**FIGURE 1:**
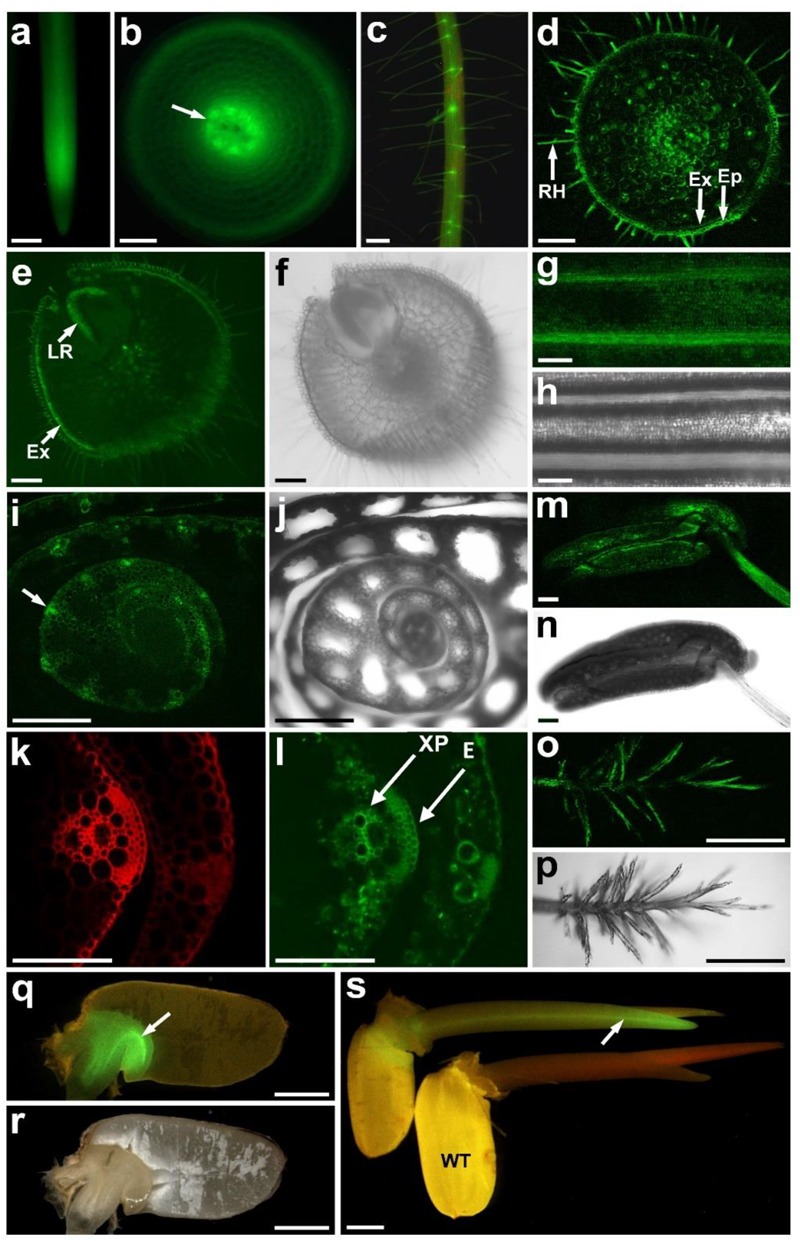
Tissue-specific expression of *OsALMT4*. Transgenic T_0_ and T_1_ rice plants transformed with GFP using the 2,496 bp region upstream of the *OsALMT4* coding region as a promoter. **(a)** GFP fluorescence in an intact root; **(b)** transverse section near the root apex showing fluorescence in the epidermis and developing stele (arrow); **(c)** mature root showing fluorescence in the epidermis and root hairs; **(d)** transverse section of a mature root showing fluorescence in the epidermis (Ep), exodermis (Ex), root hairs (RH), and stele; **(e)** transverse section showing strong fluorescence in the exodermis (Ex) and the emerging lateral root (LR); **(f)** brightfield image of **(e)**; **(g)** abaxial surface of a leaf blade showing strong GFP signals in the ribs; **(h)** brightfield image of **(g)**; **(i)** transverse section of a new tiller showing high expression in the vasculature (arrow); **(j)** brightfield image of **(i)**; **(k)** magnified image of **(i)** under UV to show the autofluorescence from the cell wall; **(l)** GFP fluorescence of the tissue in **(k)** with arrows showing the epidermis (E) and xylem parenchyma (XP); **(m)** GFP fluorescence in the stamen; **(n)** brightfield image of **(m)**; **(o)** GFP fluorescence in the stigma; **(p)** brightfield image of **(o)**; **(q)** GFP fluorescence in the scutellum (arrow) of a germinating grain; **(r)** brightfield image of **(q)**; **(s)** GFP fluorescence in the coleoptile of a transgenic (Pro4:GFP) and WT seedling. A summary of the results from T_0_ and T_1_ plants is presented in Supplementary Figure S2. Bars = 0.5 mm in **(a,c,i,j,o,p)**; 0.1 mm in **(b,d–h,k–n)**; 1.0 mm in **(q–s)**.

We showed previously that *OsALMT4* is expressed in roots and leaves of rice seedlings but details of the tissue-specific expression is unknown. To resolve this we first measured *OsALMT4* expression in roots and shoots of 5-week-old hydroponically grown plants using quantitative RT-PCR and *GAPDH* as the reference gene. The results indicated that expression in the shoots was more than 10-fold greater in the shoots than roots (0.095 ± 0.024 and 0.0098 ± 0.003, respectively). To investigate tissue-specific expression we stably transformed rice with the GFP gene driven by the *OsALMT4* promoter region (2,496 bp upstream of coding region). T_0_ plants were first transferred from tissue culture to hydroponic solution. GFP fluorescence was detected in newly emerged roots and especially nearer the apices (**Figure 1a**), perhaps due to the high cytoplasmic content of cells in that region. Transverse sections near the root apices showed fluorescence in most cells with strong signals in the developing stele (**Figure 1b**). In older plants fluorescence was detected in the epidermis and root hairs of mature seminal roots (**Figure 1c**). Transverse sections of these roots confirmed GFP expression in the epidermis, exodermis and root hairs as well as in the stele (**Figure 1d**). Strong signals also occurred at the site of lateral root emergence (**Figures 1e,f**). In leaves, fluorescence was detected in the main ribs and widely over the abaxial surface of leaf blades (**Figures 1g,h**) but not in guard cells. Fluorescence was observed in the ligule and auricle at the junction of the leaf blade and sheath (Supplementary Figures S1a–d). Widespread expression was detected in transverse sections of newly emerged tillers including in the epidermis and xylem parenchyma (**Figures 1i–l**).

In flowers, GFP fluorescence was detected in anthers (**Figures 1m,n**), in the stigma (**Figures 1o,p**), the junction between the filament and anther, as well as the lemma, palea, and sterile lemma (Supplementary Figures S1e–l). At the milk stage of grain development (∼14 days after flowering) weak fluorescence was detected on the outer side of the seed coat (Supplementary Figures S1m,n). When the seed coat was peeled back fluorescence could be detected in the aleurone layer and inner seed coat (tegumen or pericarp). Transverse sections confirmed expression in the aleurone layer and also the nucellar projection (Supplementary Figures S1o–x). Fluorescence decreased by the dough stage (28 days after flowering) but GFP signals were detected in the rachis (Supplementary Figures S1y,z).

GFP fluorescence was detected in the scutellum of embryos from T_1_ grain 2 days after imbibition (**Figures 1q,r**). No fluorescence was detected in the starchy endosperm. Five days after imbibing, GFP expression was detected in the newly emerged coleoptile, especially the vasculature (**Figure 1s** and Supplementary Figure S1a). After 10 days the first leaf had formed and GFP signals were detected at the collar, the junction between the blade and the sheath (Supplementary Figure S1b). We conclude that *OsALMT4* is expressed widely throughout the plants but not in guard cells. Supplementary Figure S2 summaries the results of the tissue-specific expression presented in **Figure 1** and Supplementary Figure S1.

### Responses of *OsALMT4* Expression to Abiotic Stresses

**FIGURE 2:**
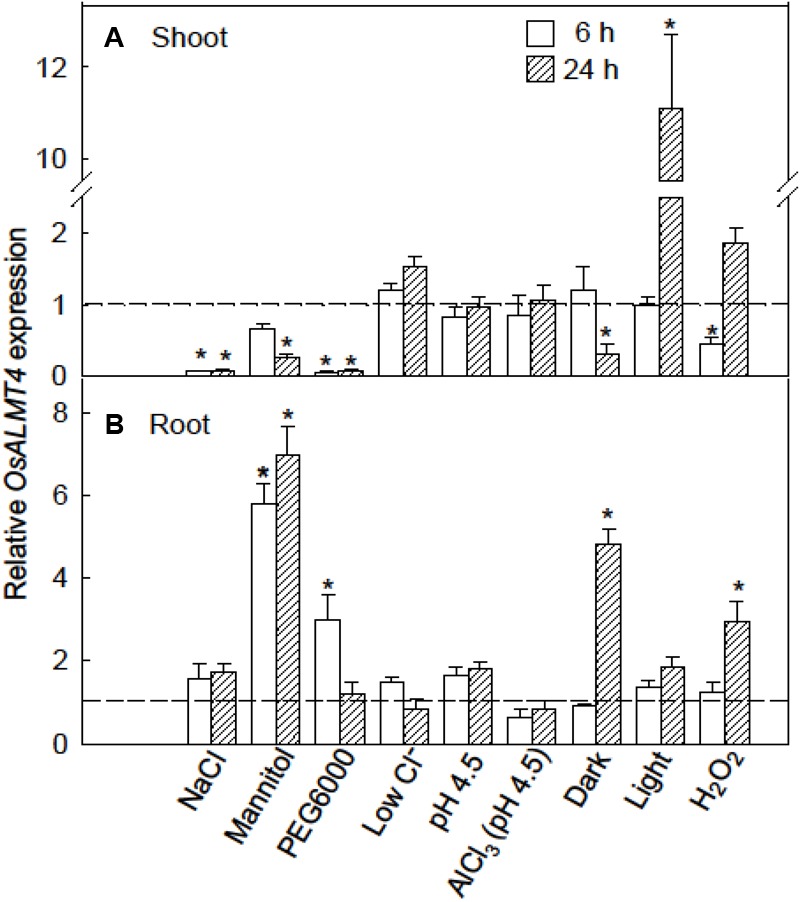
Response of OsALMT4 expression to abiotic stresses. *OsALMT4* expression in the shoot **(A)** and root **(B)** tissue of wild-type rice seedlings was measured using qRT-PCR after 6 and 24 h exposure to various treatments added to the nutrient solution including 200 mM NaCl, 350 mM mannitol (same osmolarity as the NaCl treatment), a low-Cl^-^ solution, and 300 μM H_2_O_2_. Other treatments were low pH (pH 4.5) with or without 100 μM AlCl_3_, and either continual light (∼500 μmol m^-2^s^-1^) or continual darkness. The dashed line shows qRT-PCR data collected at the same time from control plants grown in parallel in standard hydroponic treatments. Expression measurements used two reference genes (*GAPDH* and *eEF-1α*) combined to provide a single result according to the software in Bio-Rad CFX Manager^TM^. Data show mean and SE from three biological replicates where each replicate is the mean of three technical replicates. An asterisk indicates a significant difference (*P* < 0.05) between the treatment and control at the same time using a *t*-test.

We hypothesized that *OsALMT4* expression would change in response to conditions that are likely to increase membrane transport activity such as changes in ionic environment, osmotic stress and light intensity. To test this we monitored *OsALMT4* expression levels in the shoots and roots of WT plants after a 6 and 24 h exposure to various treatments. Plants were grown in hydroponics and treatments were directly compared with control plants grown in parallel in standard conditions. The results shown in **Figure 2** are summarized in Supplementary Figure S3. Osmotic stress imposed by 200 mM NaCl, 20% PEG6000 or 350 mM mannitol decreased *OsALMT4* expression in the shoots (**Figure 2A**). Small decreases also followed prolonged darkness and 6 h with hydrogen peroxide while expression in shoots increased 10-fold after 24 h of constant light. In roots, *OsALMT4* expression was increased by mannitol, PEG6000, prolonged darkness and hydrogen peroxide (**Figure 2B**).

### Responses of *OsALMT4* Expression to Hormones and GABA

**FIGURE 3:**
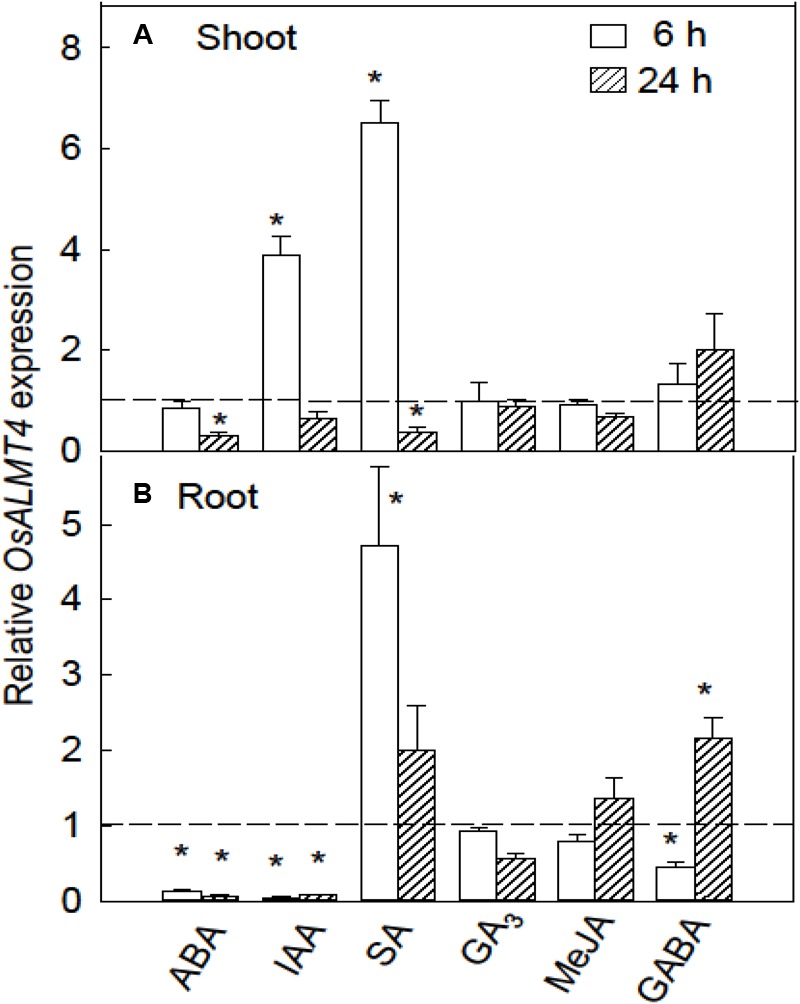
Response of *OsALMT4* expression to hormones and GABA. *OsALMT4* expression was measured in shoots **(A)** and roots **(B)** after exposure to 100 μM of abscisic acid, gibberellin (GA_3_), indole acetic acid (IAA), salicylic acid (SA), and methyl jasmonic acid (MeJA) or to 1 mM γ-amino butyric acid (GABA). The dashed line shows qRT-PCR data collected at the same time from control plants grown in parallel in standard hydroponic treatments. Expression measurements used two reference genes (*GAPDH* and *eEF-1α*) combined to provide a single result according to the software in Bio-Rad CFX Manager^TM^. Data show mean and SE from three biological replicates where each replicate is the mean of three technical replicates. An asterisk indicates a significant difference (*P* < 0.05) between the treatment and control using a *t*-test.

*OsALMT4* expression was monitored in WT plants during treatment with hormones including indole acetic acid (IAA), ABA, gibberellin (GA_3_), methyl jasmonate (MA) as well as with γ-amino butyric acid (GABA). After 7 days in hydroponics seedlings were transferred to various treatments and roots and shoots harvested after 6 and 24 h. The hormones had contrasting effects on *OsALMT4* expression in the shoots. IAA and SA strongly increased expression in shoots after 6 h while longer treatments with ABA and SA slightly reduced *OsALMT4* expression compared to controls (**Figure 3A**). ABA and IAA caused large and sustained decreases in roots while 6 h in SA increased expression more than fourfold. Treatment with 1 mM GABA had no effect on expression in shoots but caused small variable changes in the roots (**Figure 3B**).

### Characterizing Malate Efflux From Transgenic Rice With Increased *OsALMT4* Expression

We previously generated transgenic rice lines (cv. Nipponbare) with altered expression of *OsALMT4* (Liu et al., 2017). Independent homozygous lines constitutively over-expressing *OsALMT4* (OX2 and OX5) had 20- to 40-fold higher *OsALMT4* expression levels than their null sister lines (OX2_null and OX5_null) while two independent homozygous RNAi lines (R24 and R58) had expression reduced to 10–20% of null lines (R24_null and R58_null) (Liu et al., 2017). All the OX and RNAi lines were fertile, showed similar root/shoot ratios, leaf chlorophyll content and tiller numbers as their nulls except the OX lines tended to accumulate less biomass (Liu et al., 2017). The independent OX lines also showed greater malate concentrations in the xylem sap and a constitutive release of malate from their roots which was not detected in the null lines or WT plants (Liu et al., 2017). These fluxes varied from 3 to 12 nmol seedling^-1^ h^-1^ depending on seedling age and the number of roots they had. These rates were comparable to those measured from aluminum-resistant wheat plants (Ryan et al., 1995).

**FIGURE 4:**
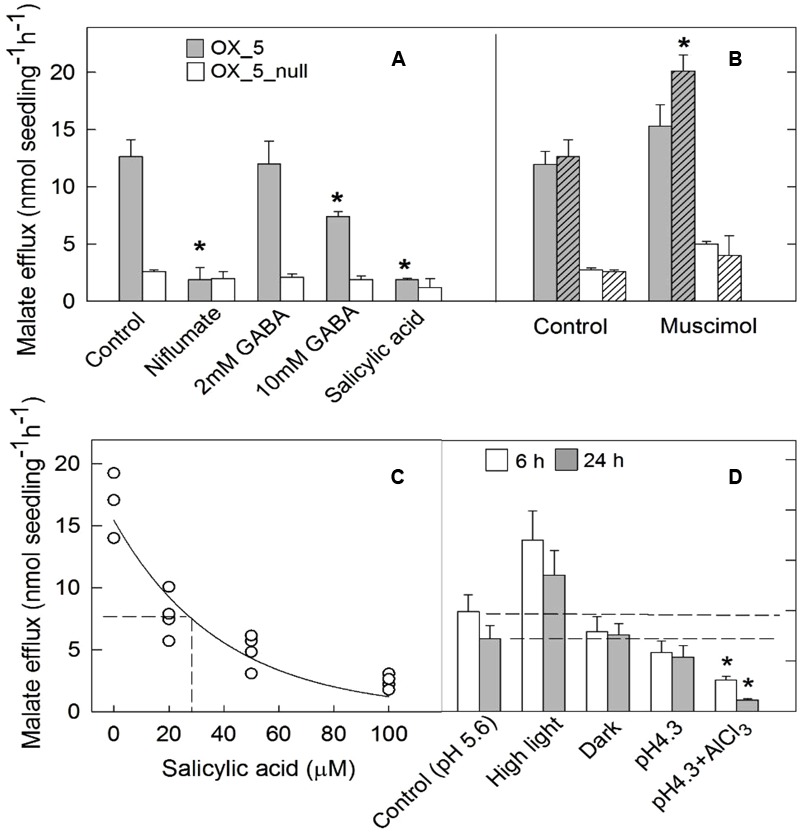
Characterization of malate efflux from OX lines. Malate efflux from transgenic and null plants was measured after exposure to various treatments. Seedlings were grown in flasks with sterile nutrient solution (pH 5.6) and placed in a growth room with 50 μmol m^-2^s^-1^ light intensity. After 5 days the solution was replaced with 10 ml of 0.5 mM CaCl_2_ (pH 5.6) and subject to different treatments. **(A)** Malate efflux from OX5 and OX5_null lines measured after 20 h in control solution, or with added 100 μM niflumate, 2 mM or 10 mM GABA or 100 μM salicylic acid. **(B)** Results from two separate experiments (hatched and unhatched) showing malate efflux from OX5 and OX5_null plants after 20 h in control solution or 100 μM muscimol. **(C)** Malate efflux from OX5 plants after 6 h in different salicylic acid concentrations. **(D)** Malate efflux measured after 6 and 20 h treatment in constant darkness, constant higher light intensity (550 μmol m^-2^s^-1^), low pH solution (pH 4.3) and low pH with 100 μM AlCl_3_. Data are mean and SE (*n* = 3 or 4). An asterisk indicates a significant difference (*t*-test; *P* < 0.05) between the control and treatment within each line using a *t*-test.

We measured the effects of various treatments on malate efflux from the OX lines. Seedlings were grown for 4 days in flasks with sterile hydroponic solution at low ambient light (50 μmol m^-2^s^-1^). The solution were replenished and malate release was measured after 6 h and 24 h in various treatments as shown in **Figure 4**. Niflumate is a known antagonist of anion channels which has been shown to inhibit the activity of other members of the ALMT family (Zhang et al., 2008). Niflumate effectively inhibited malate efflux from OX5 plants to less than 20% of controls (**Figure 4A**). Treatment with 2 mM GABA had no effect on malate efflux but 10 mM GABA decreased efflux by almost half. SA effectively inhibited malate efflux to 18% of controls after 6 h. In a separate experiment the half maximum inhibition (*K*_i_) for SA was determined to be 25 μM (**Figure 4C**). No consistent changes were found with the other hormones (Supplementary Figure S4). Muscimol is an agonist of GABA_A_ receptors in animal cells. In two separate experiments treatment with 100 μM muscimol increased malate efflux from OX5 plants by 60% in one but not the other (**Figure 4B**). This small variation is likely due to differences in the number of roots on each seedling. The important finding is that GABA and muscimol are not having the large and consistent effect on malate efflux reported for other ALMTs.

Exposure to a constant light intensity of 550 μmol m^-2^s^-1^ tended to increase malate efflux from the OX plants but the changes were not significantly different from controls (**Figure 4D**). Continual darkness had no effect on malate efflux which is consistent with the low light levels in control conditions. Reducing solution pH from 5.6 to 4.3 had no effect on malate efflux but addition of 100 μM AlCl_3_ (pH 4.3) decreased efflux after 6 and 24 h.

To determine whether the organic anion release from transgenic plants was associated with changes in pH adjacent to the roots, 7-day-old seedlings were transferred to shallow dishes with a mixture of agar and pH indicator bromocresol purple (∼pH 6.0) and monitored over 6 h. No consistent differences in color change were detected around the roots or root apices of the transgenic and null plants (data not shown).

### Growth of OX Lines in Different Abiotic Stresses

**FIGURE 5:**
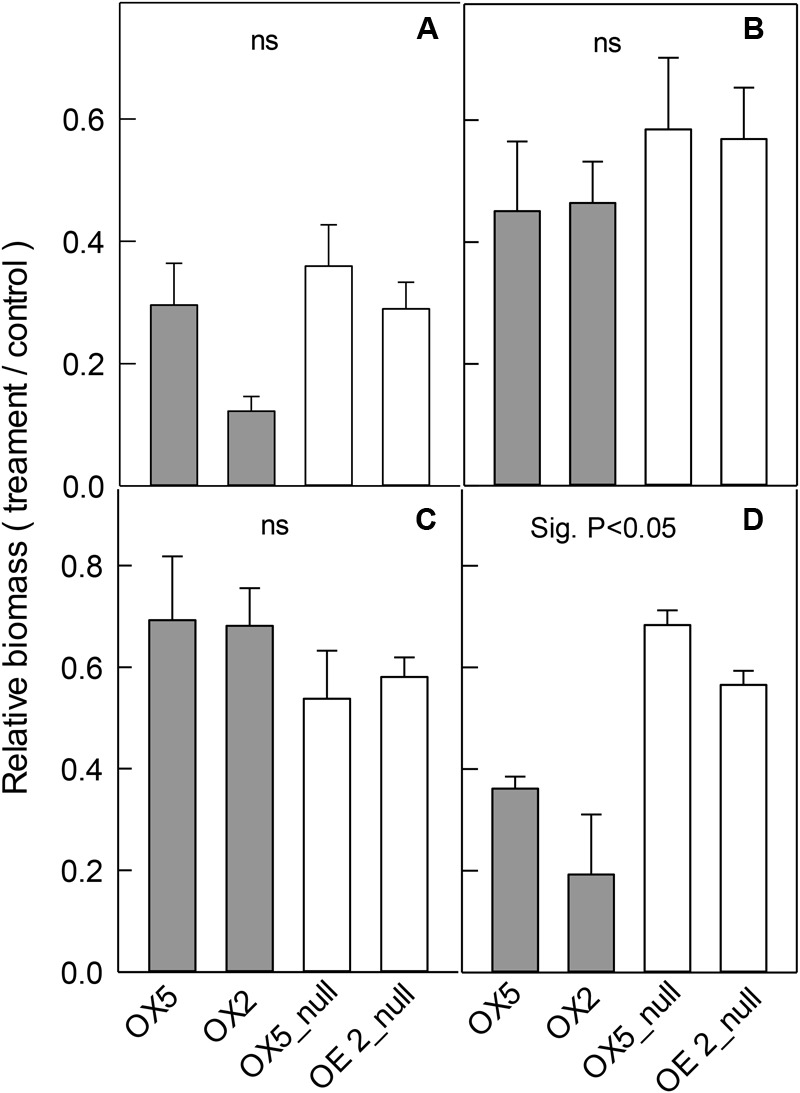
Effect of abiotic stresses on the growth of OX lines. Growth of transgenic lines with increased *OsALMT4* expression (OX5 and OX2) and null lines (OX5_null and OX2_null) was measured after longer-term treatments in various abiotic stresses. Data show the relative growth (biomass in treatment/biomass in control) for each line after 28 days in a growth chamber in hydroponics with and without **(A)** 100 mM NaCl, **(B)** 50 mM mannitol or **(C)** low-Cl^-^ solution. In separate experiments shown in **(D)** plants were grown for 56 days in soil in growth chambers where the maximum diurnal light intensity was either 700 μmol m^-2^s^-1^ (control) or 300 μmol m^-2^s^-1^ (low). Results show total biomass in **(A–C)** whereas shoot biomass is shown in **(D)**. Results show mean and SE (*n* = 4–6). Biomass data is shown in Supplementary Table S1. The combined data from the RNAi lines were significantly different from the combined data from the null lines for the low light treatment only (*P* < 0.05) using a *t*-test test as shown.

*OsALMT4* expression was affected by various abiotic treatments therefore we hypothesized that altering *OsALMT4* expression would affect plant growth during longer-term exposures to these stresses. We tested this in the OX lines first by growing independent transgenic lines and their null lines for 28 days in hydroponics with 100 mM NaCl, 50 mM mannitol, low-Cl^-^ solution or in pots under high and low light intensities (**Figure 5**). Since growth of the transgenic lines sometimes differed in control conditions (Liu et al., 2017), comparisons between each transgenic and related null line were based on relative changes in biomass which is defined as the ratio (biomass in treatment/biomass in the control). All the treatments reduced total plant biomass by 40–60% relative to controls (Supplementary Table S1). Relative biomass in the OX was similar to the null lines in all cases except for one: biomass of OX2 was reduced more by high salt than its null line (**Figure 5A**). Apart from this single difference, the results indicate that increasing *OsALMT4* expression did not consistently alter plant growth in the high salt, mannitol or in a low-Cl^-^ treatment (**Figures 5A–C**).

Lastly we tested the effect of light intensity on plant growth. OX5 and OX2 plants and their nulls were grown in chambers with either a control light intensity (700 μmol m^-2^s^-1^) or a low light intensity (300 μmol m^-2^s^-1^). The low light treatment significantly decreased total plant biomass of all lines (Supplementary Table S1) but the relative biomass (biomass at low light/biomass at high light) for both OX lines was significantly smaller than their nulls. For example, relative biomass of OX5 was 35% whereas for OX5_null it was 68% (**Figure 5D**).

### Growth of RNAi Lines in Different Abiotic Stresses

**FIGURE 6:**
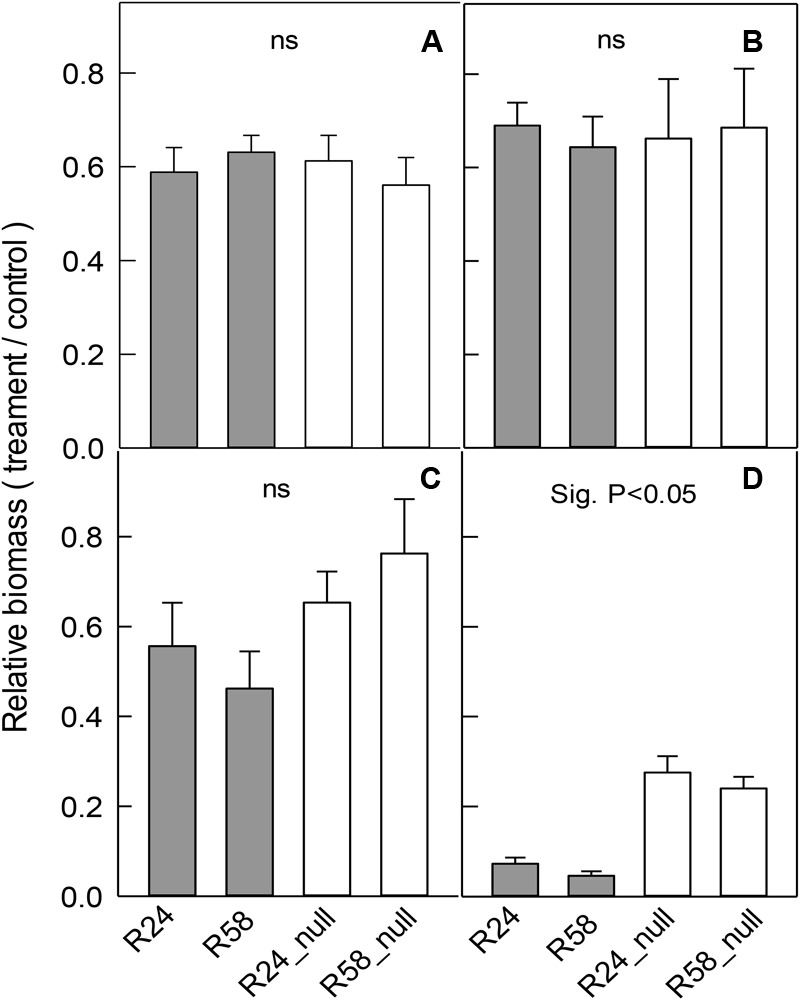
Effect of abiotic stresses on the growth of RNAi lines. Growth of RNAi lines with reduced *OsALMT4* expression (R24 and R58) and null lines (R24_null and R58_null) was measured after longer-term treatments in different abiotic stresses. Data show the relative growth (biomass in treatment/biomass in control) for each line after 28 days in a growth chamber in hydroponics with and without **(A)** 100 mM NaCl, **(B)** 50 mM mannitol, or **(C)** low-Cl^-^ solution. In a separate experiments shown in **(D)** plants were grown for 28 days in soil in growth chambers where the maximum diurnal light intensity was either 700 μmol m^-2^s^-1^ (control) or 300 μmol m^-2^s^-1^ (low). Total biomass is shown in **(A–C)** whereas shoot biomass is shown in **(D)**. Results show mean and SE (*n* = 4–6). Biomass data is shown in Supplementary Table S1. The combined data from the RNAi lines were significantly different from the combined data from the null lines for the low light treatment only (*P* < 0.05) using a *t*-test as shown.

Independent RNAi lines, R58 and R24, and their nulls were grown in the same treatments as above. The salt, mannitol and low-Cl^-^ treatments reduced total plant biomass and the relative changes in biomass were similar in each RNAi line compared to their null line (**Figures 6A–C**). These results indicate that reducing *OsALMT4* expression to ∼15% of null lines did not significantly alter the growth of rice to these stresses.

In separate experiments, the RNAi lines were grown in the high (control) and low light intensity treatments. Low light significantly decreased total biomass of all lines (Supplementary Table S1) as before but the relative decreases in R58 and R24 were four to fivefold greater than the relative decreases in R58_null and R24_null lines (**Figure 6D**).

### Photosynthetic Capacity of the OX and RNAi Lines

The growth experiments above indicated that growth of rice to grow in a reduced light environment was compromised when *OsALMT4* expression was either increased or reduced. One possible explanation for the sensitivity of the OX and RNAi lines to low light is that the photosynthesis is affected by the altered malate fluxes. We tested this directly by measuring the total sugar content of these plants and their photosynthetic capacity. Metabolomics analysis of sugars was performed on shoot and root tissues collected from transgenic and null lines grown in hydroponics. A total of 23 sugars were detected in the shoots and 18 in the roots but no significant differences were detected between the OX and RNAi lines and their nulls (Supplementary Table S2). The response of CO_2_ assimilation rates (*A*) to light intensity was then measured in the flag leaves of the OX and RNAi lines with the LICOR LI-6400 and compared with their null lines grown side-by-side in the same conditions. The results indicate that photosynthetic rates at 250 and 1500 μmol m^-2^s^-1^ light intensity was not significantly different between the transgenic and null lines. Similarly, no differences were detected in maximum quantum yield, convexity factor or stomatal conductance to water vapor (*g*_s_) at low irradiance or saturating irradiance. The estimated theoretical maximum rate of electron transport at saturating light (*J*_max_) was as estimated from the light curves using the online tool by Sharkey et al. (2007) based on the model of photosynthesis described previously (Farquhar et al., 1980). The results were also similar in the transgenic and null lines for the OX lines and RNAi lines (**Table 1** and Supplementary Figure S5).

**Table 1:** Photosynthetic parameters of rice with altered *OsALMT1* expression.

Parameters Light		Measured 1500 (μmol m^-2^s^-1^)>		250 (μmol m^-2^s^-1^)		Derived		
[CO_2_]		400 (ppm)		400 (ppm)				
	SPAD	*A*	*g*_s_	*A*	*g*_s_	Φ	𝜃	*J*_max_
OX5_null	30.8 ± 1.5	22.9 ± 5.7	0.49 ± 0.22	11.2 ± 0.8	0.35 ± 0.84	0.32 ± 0.01	0.78 ± 0.07	162 ± 40
OX5	32.4 ± 0.3	17.5 ± 4.1	0.35 ± 0.10	9.7 ± 1.3	0.28 ± 0.83	0.30 ± 0.07	0.85 ± 0.09	117 ± 29
R58_null	30.7 ± 0.9	20.0 ± 6.1	0.41 ± 0.13	10.0 ± 1.3	0.32 ± 0.84	0.30 ± 0.02	0.77 ± 0.12	141 ± 43
R58	31.1 ± 0.4	24.8 ± 5.3	0.60 ± 0.30	11.1 ± 0.5	0.37 ± 0.83	0.33 ± 0.03	0.79 ± 0.05	176 ± 39

*Standard photosynthetic parameters were measured on OX5 plants with increased *OsALMT4* expression and R58 plants with reduced *OsALMT4* expression and their respective null lines. Average of CO_*2*_ assimilation rate (*A*,μmol CO_*2*_ m^-*2*^s^-*1*^) and stomatal conductance (*g*_*s*_, mol H_*2*_O m^-*2*^s^-*1*^) from the light response curves is presented as well as biochemical derived parameters (Φ, quantum yield; 𝜃, convexity factor; *J*_*max*_, theoretical maximum rate of electron transport at saturating light). Gas exchange was measured for the flag leaves of 6-week-old plants grown in a glasshouse (*n* = 4 biological replicates per genotype). Irradiance intensity and [CO_*2*_] are indicated. One-way analysis of variance (ANOVA) using a general linear model was performed using the function *aov* in R language to quantify the effects of genotype on different parameters. Differences between means in a pairwise comparison were tested using Tukey’s honestly test with package “predictmeans” (*n* = 4) and no significant differences were detected between OX5 and OX5_null or between R58 and R58_null.*

## Discussion

We investigated the function of *OsALMT4* and found that it is widely expressed in roots and shoots but not guard cells. Expression was especially clear in the vasculature, flowers, developing grain and at the collar (junction between the leaf blade and the sheath). All these tissues have high metabolic and transport activity which are either sinks for nutrients or important for nutrient transfer. Movement of ions and even non-charged nutrients, such as sugars, across membranes can be electrogenic (affects the membrane potential difference) since the neutral molecules are often accompanied by protons. This requires the counter-transport of other ions to maintain a stable membrane potential and to help regulate pH. For instance, *OsALMT4* is expressed in the nucellar projection of the developing grain that coordinates the movement of nutrients from the vascular to the endosperm transfer cells (Thiel et al., 2008). The function of *OsALMT4* in this tissue could be related to nutrient transfer whereby malate transport balances the unloading of nutrients to the developing endosperm. The high *OsALMT4* expression in the vascular tissue and collar is also consistent with this idea. The collar contains the nodal tissues which distribute nutrients to stem or leaves via interacting vascular systems (Yamaji and Ma, 2014). Mineral elements need to be transferred between different vascular bundles in the nodes to preferentially distribute ions to developing tissues. This is controlled by transporters localized to these nodal cells and the movement of charged solutes also need to be balanced by the movement of other ions. A similar function assisting nutrient distribution was proposed for ZmALMT1 in maize but this channel protein was shown to be more permeable to inorganic anions than organic anions (Pineros et al., 2008; Sharma et al., 2016).

OsALMT4 function was investigated by monitoring transcript levels in WT plants under various treatments. An interaction was revealed between the plant hormone SA and the expression and function of OsALMT4. SA induced expression in roots and shoots by fivefold or sixfold within 6 h. We also found that SA treatment effectively inhibited malate efflux via OsALMT4 in the transgenic OX lines with a *K*_i_ of 25 μM indicating that SA could affect protein function as well as expression. SA-induced small changes in the expression of another member of the family, *AtALMT1*, in Arabidopsis (Kobayashi et al., 2013) but no attempts were made to test protein function in that study. SA is known to be involved in many aspects of plant development, pathogenesis and systemic acquired resistance. It can also affect stomatal aperture by modulating membrane transporters, possibly via the production of reactive oxygen species (Gaffney et al., 1993; Hayat and Ahmed, 2007; Khokon et al., 2011). We speculate that some SA-mediated signals in rice might depend on altering ion fluxes across the plasma membrane. The changes in malate efflux could represent a chemical signal or, by affecting membrane potential, represent an electrical signal. It will be interesting to investigate this further with electrophysiological approaches and to also to determine whether OsALMT4, and perhaps other ALMTs, contribute to the SA-related signaling network.

Other hormones caused variable effects on *OsALMT4* expression. Methyl jasmonate and gibberellin had no effect on expression whereas abscisic acid and auxin caused large and sustained reductions in expression in roots and auxin increased expression in shoots. These results contrast with those reported for Arabidopsis where the expression of *AtALMT1*, was induced by ABA and IAA (Kobayashi et al., 2013). Therefore, the expression of different *ALMT*s to hormones can be quite different. A recent study proposed that ALMT proteins function as GABA receptors in plants that transduce chemical signals into electrical signals by modulating anion fluxes across membranes (Ramesh et al., 2015). That study examined TaALMT1 function in wheat plants as well as a range of other ALMTs expressed in *Xenopus* oocytes. The transport activity for all of them was inhibited by 70–80% with millimolar concentrations of GABA and micromolar concentrations of muscimol (an agonist of GABA_A_ receptors in animal cells). In the present study, GABA had no effect on *OsALMT4* expression in the shoots and only small variable changes in the roots. At 10 mM, GABA inhibited malate efflux in the OX lines by less than 50% whereas muscimol either had no effect or caused a small increase in efflux. While significant, the magnitude of these changes are much smaller than the inhibition reported for other ALMTs tested and indicates these compounds are having little direct effect on OsALMT4 function. The OsALMT4 protein possesses the same putative binding motif for GABA as the other ALMTs so further work is required to determine why OsALMT4 responds differently to the others. Perhaps the regulatory interactions between GABA and OsALMT4 are different in rice plants or perhaps only a subset of ALMT proteins function as GABA receptors in the way described by Ramesh et al. (2015).

Several abiotic stresses affected *OsALMT4* expression and some of the largest changes were associated with ionic and osmotic stress. NaCl and mannitol treatments reduced *OsALMT4* expression in the shoots while mannitol increased expression in the roots. These changes likely reflect complex interactions between stress signals and *OsALMT4* expression which would modulate anion transport and possibly osmotic gradients, water movement and turgor. The decreased expression of *OsALMT4* in shoots during osmotic stress could reduce ion loss from leaf cells and contribute to the maintenance of leaf turgor. We tested this link with longer-term growth experiments in high salt and high mannitol treatments but the changes in biomass of the OX lines and RNAi lines were not significantly different from their null lines over this time period. It is possible that longer treatments or higher concentrations of salts might generate a difference but, even if OsALMT4 is involved in these responses, it is very likely that other transport processes will compensate for the changes in OsALMT4 activity.

OX and RNAi plants were also grown in low-Cl^-^ conditions for several weeks to test whether removal of this major inorganic anion increased the contribution of other transporters (e.g., OsALMT4) that move organic anions across membranes – especially abundant anions like malate. However, no significant differences were detected in the biomass between the OX and null lines or the RNAi and null lines (**Figures 5, 6**). Other anions and transport processes may well compensate for the reduced Cl^-^ availability.

Different light environments altered *OsALMT4* expression so the effect of light intensity on plant growth was also measured over a longer period. Relative biomass accumulation in both the OX and RNAi lines was significantly smaller than nulls under lower light intensity compared to the high light intensity. In other words, altering *OsALMT4* expression, either up or down, affected rice growth in low light. It is unclear what causes these effects but we demonstrated that it is not related to changes in total sugar concentrations in the plants or photosynthetic capacity. Indeed, the response of CO_2_ assimilation to increasing light intensity and stomatal conductance, *g*_s_, were not significantly different between the transgenic lines and their nulls. Instead, we propose that the reduced growth at low light may be linked to the disruption of malate transport and altered carbon compartmentation within the tissues of the transgenic lines as explained below. A previous study showed that total malate concentrations were not perturbed in shoots of the transgenic lines (Liu et al., 2017). However, the concentration of malate in the xylem sap was greater in the OX lines compared to nulls and the compartmentation of manganese was also altered (Liu et al., 2017). Those results are consistent with changes to *OsALMT4* expression affecting the distribution of malate between the apoplast and symplast and ultimately changing malate compartmentation within tissues. Malate is among the most prevalent anions in plants participating in key anabolic and catabolic pathways. It is an important part of the tricarboxylic acid cycle, glyoxylate cycle, C4 photosynthetic carbon reduction, crassulacean acid metabolism and contributes to nutrient acquisition (Ryan et al., 2001), osmotic adjustment (Fernie and Martinoia, 2009) electroneutrality (Osmond, 1976; Ryan et al., 2001; Roelfsema and Hedrich, 2005) and cytosolic pH regulation (Sakano, 1998; Kovermann et al., 2007). Some carboxylates have other important metabolic influences as well, beyond being a common intermediate. For instance, apart from being important metabolites, malate and citrate can also act as signaling molecules which directly affect transport activities, metabolism, photosynthesis, and gene expression (Hedrich and Marten, 1993; Nunes-Nesi et al., 2005; Meyer et al., 2010a; Finkemeier et al., 2013). The altered fluxes of malate across membranes, either increased or decreased, will affect carbon partitioning, disrupt signaling pathways and influence nutrient movement – especially when carbon fixation and energy levels are reduced by a low-light environment. All of these could affect cellular metabolism and growth.

OsALMT4 is the first ALMT from rice to be examined in detail and the first member of clade 5 in the protein family to be characterized from any species (Liu et al., 2017). Therefore no direct comparisons can be made with other genes in this clade. Clade 1, by comparison, contains several ALMTs that have been studied including HvALMT1 from barley, TaALMT1 from wheat and ZmALMT1 from maize and OsALMT4 shares some features with these. For instance, TaALMT1 and HvALMT1 both function as malate-permeable channels and *HvALMT1* is expressed widely throughout the plant like *OsALMT4* (Gruber et al., 2010, 2011; Xu et al., 2015). The Arabidopsis protein AtALMT12, in clade 3, reportedly generates the rapid or R-type/QUAC anion currents well-characterized in plants (Meyer et al., 2010b). These currents were first identified in guard cells of *Vicia faba* (Schroeder and Keller, 1992) but have now been observed in other cell-types including roots (Diatloff et al., 2010). Proposed functions of R-type/QUAC channels include the regulation of stomatal aperture, plant defense, cell signaling and generation of action potentials, chemical homeostasis and organic anion release from roots. The activity of R-type/QUAC channels can be modulated by cytosolic nucleotides, calcium, anion gradients, sulfate concentration, phosphorylation, ABA, and H_2_O_2_ but this varies between proteins (Roberts, 2006; Diatloff et al., 2010). The channel antagonist niflumic acid inhibits many R-type/QUAC channels and we showed here that malate efflux via OsALMT4 is also inhibited by niflumic acid. Further electrophysiological studies are required to test whether all members of the ALMT family, including OsALMT4, show characteristics of R-type/QUAC channels.

## Conclusion

This study characterized Os*ALMT4* gene in rice first by showing it is expressed widely and especially in tissues with high metabolic and transport activity such as the vasculature, emerging lateral roots, flowers and developing grain. *OsALMT4* expression was shown to respond to certain environmental signals including osmotic stress, light intensity and the hormones ABA, IAA, and SA. Furthermore, SA effectively inhibited OsALMT4 function in transgenic lines. These results suggest OsALMT4 performs various functions in different tissues by facilitating malate efflux from cells. Furthermore, changing *OsALMT4* expression, either up or down, in transgenic rice plants compromised growth in a lower light environment which we attribute to the alterations in malate transport.

## Author Contributions

JL assisted in the design and performed most experiments, analyzed the data, and assisted with writing the article. MX provided technical support and experimental advice. ED assisted in experimental design and assisted with writing the article. GE coordinated the photosynthetic experiments and analyzed that data. MZ complemented the writing. RW supervised the microscopy and gave technical assistance. PR conceived the project, assisted in experimental design, and coordinated writing of the article. PR, ED, and MZ supervised JL.

## Conflict of Interest Statement

The authors declare that the research was conducted in the absence of any commercial or financial relationships that could be construed as a potential conflict of interest.
