# Supplementary material for: Altered Expression of the Malate-Permeable Anion Channel OsALMT4 Reduces the Growth of Rice Under Low Radiance
Source: Front Plant Sci. 2018 May 3;9:542. doi: 10.3389/fpls.2018.00542 (PMC5943490; doi:10.3389/fpls.2018.00542)
Supplement: Supplementary file 1 [file Data_Sheet_1.pdf]

OsALMT4 encodes a malate-permeable anion channel

by Liu et al

SUPPLEMENTARY FIGURES

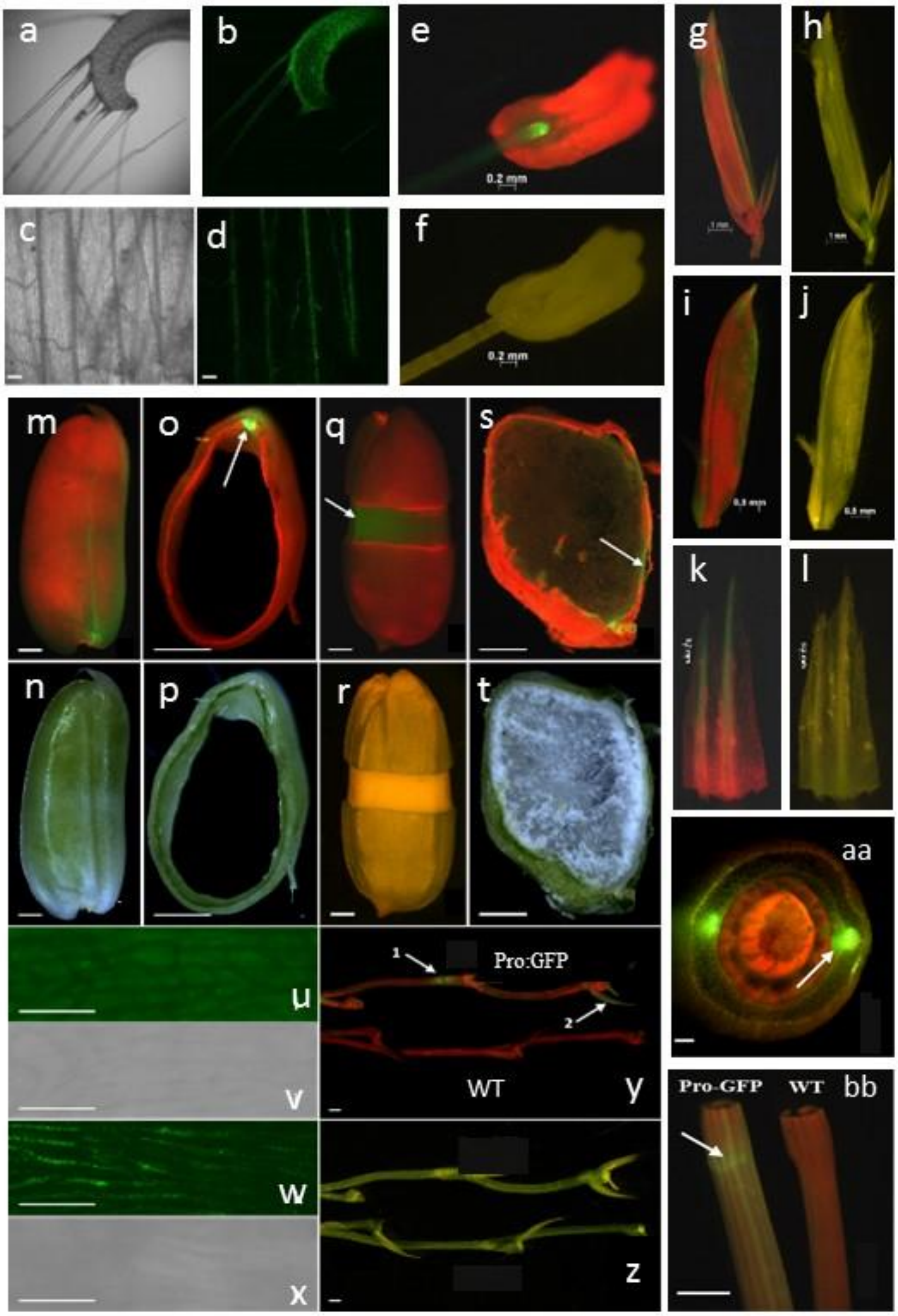

Figure S1

Figure S1 Tissue-specific expression of *OsALMT4*

Rice was stably transformed with GFP driven by the *OsALMT4* promoter (2496 bp of the DNA sequence upstream of coding region). (a) Bright-field image of the auricle; (b) fluorescent image of auricle shown in a; (c) bright-field image of the ligule tissue; (d) fluorescent image of the ligule shown in c; (e) fluorescent image of a stamen showing high expression in the filament at the junction with the anther; (f) Bright-field image of the image in e; (g) fluorescent image of a lemma; (h) bright-field image of the image in g; (i) fluorescent image of a palea; (j) bright-field image of the image in i; (k) fluorescent image of a sterile lemma; (l) bright-field image of the image in k; (m) fluorescent image of grain at the milk stage; (n) bright-field image of the image in m; (o) fluorescent image of a transverse section through grain at the milk stage showing high expression in the nucellar projection (arrow); (p) Bright-field image of the image in o; (q) fluorescent image of the dough stage of grain development showing GFP signals in the aleurone layer when part of the seed coat was peeled back (arrow); (r) bright-field image of the tissue in q; (s) fluorescent image of a transverse section through grain at the dough stage showing GFP signals in the aleurone layer (arrow); (t) bright-field image of the tissue in s; (u) fluorescent image of the aleurone layer with a confocal microscope; (v) bright-field image of the tissue in u; (w) fluorescent image of the inner seed coat with a confocal microscope; (x) bright-field image of the same tissue in w; (y) fluorescence images of a rachis from a transgenic (Pro:GFP) and WT plant showing GFP signals (arrows) in the transgenic only; (z) bright-field image of the same tissue in y; (aa) fluorescent image of a transverse section of newly emerged shoot nine days after germination showing high expression in the vascular bundle of the coleoptiles (arrow); (bb) florescent image of the stem of transgenic (Pro:GFP) and WT plants showing GFP expression in the collar (arrow) of the transgenic plant only. Scale bar in bb = 1 mm; m, n , o, p, q, r, s, t = 500 µm; c,d = 100 µm, u, v, w, x, aa = 50 µm.

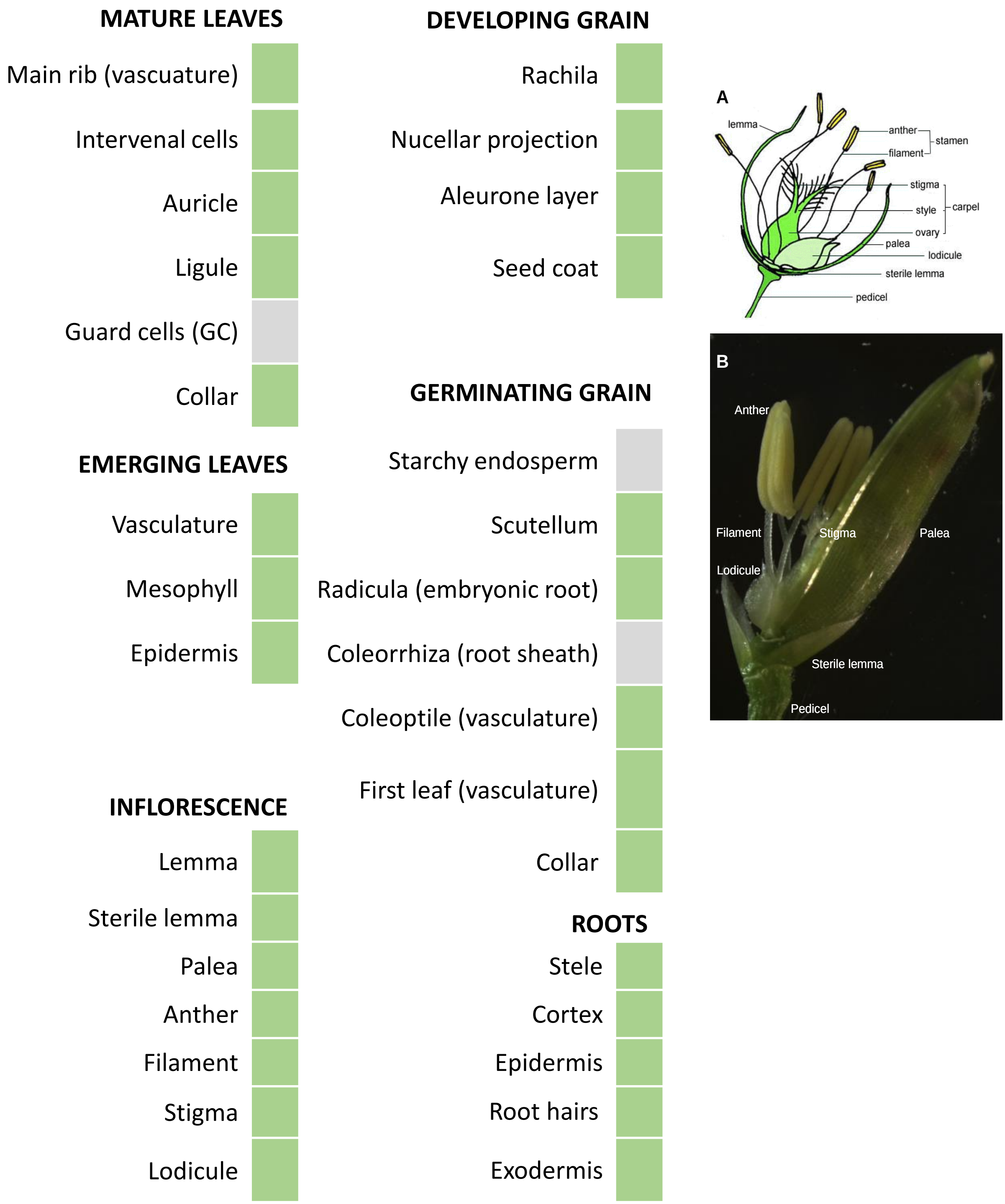

MATURE LEAVES
Main rib (vascuature)
Intervenal cells
Auricle
Ligule
Guard cells (GC)
Collar
EMERGING LEAVES
Vasculature
Mesophyll
Epidermis
INFLORESCENCE
Lemma
Sterile lemma
Palea
Anther
Filament
Stigma
Lodicule
DEVELOPING GRAIN
Rachila
Nucellar projection
Aleurone layer
Seed coat
GERMINATING GRAIN
Starchy endosperm
Scutellum
Radicula (embryonic root)
Coleorrhiza (root sheath)
Coleoptile (vasculature)
First leaf (vasculature)
Collar
ROOTS
Stele
Cortex
Epidermis
Root hairs
Exodermis
A
lemma
anther
filament
stamen
stigma
style
ovary
carpel
palea
lodicule
sterile lemma
pedicel
B
Anther
Filament
Stigma
Palea
Lodicule
Sterile lemma
Pedicel

Figure S2

Figure S2 Summary of tissue-specific expression of *OsALMT4*

Summary of the tissue specific expression of the *OsALMT4* gene in different tissues. The 2496 bp region upstream of the *OsALMT4* transcription start site was used to drive GFP expression. Green indicates expression was detected in the tissues while grey indicates no expression was detected. GC refers to guard cells. Also included are diagrams of the anatomy of rice flowers (from http://www-plb.ucdavis.edu/labs/rost/rice/reproduction/flower/flower.html)

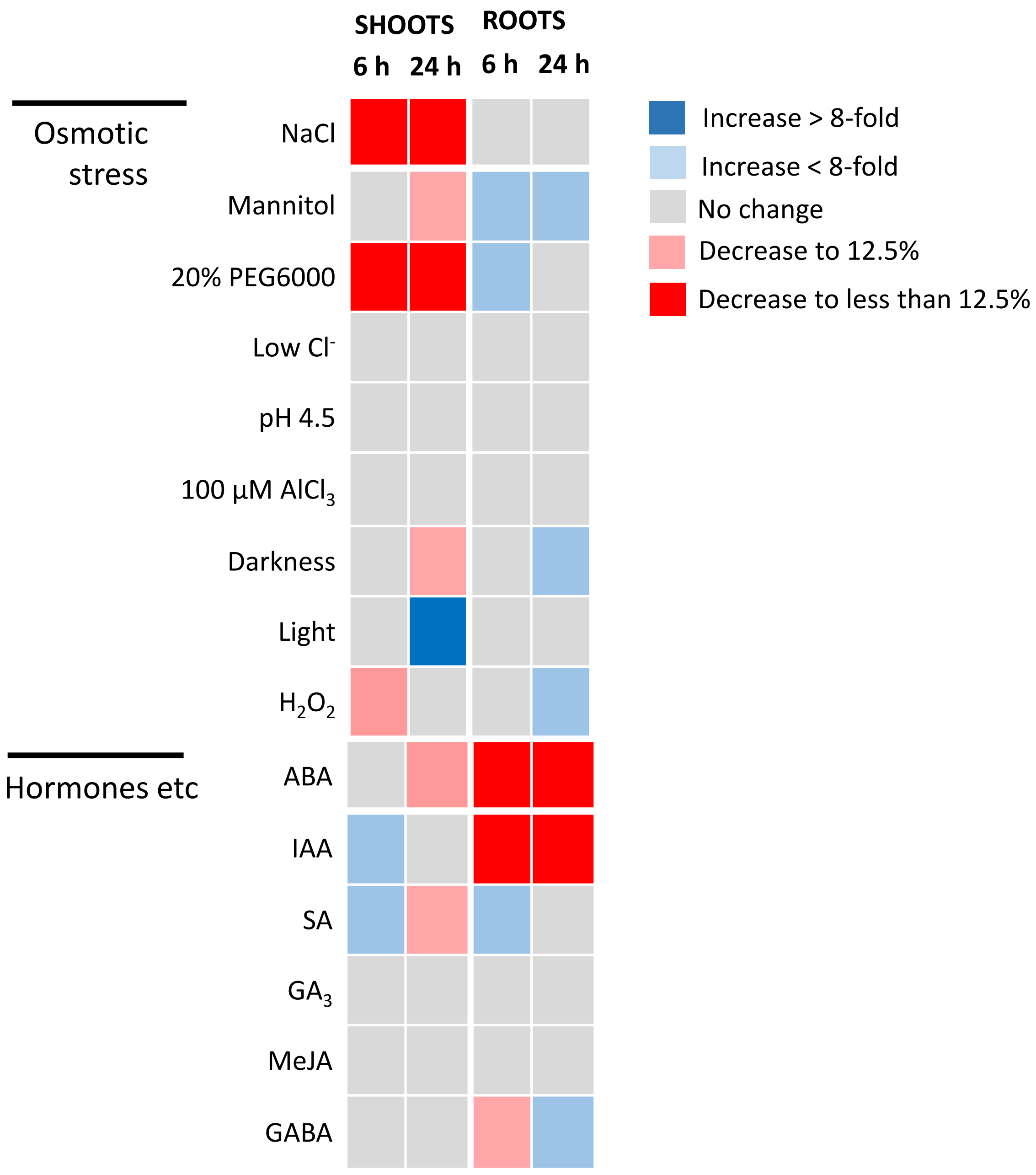
SHOOTS
ROOTS
6 h
24 h
6 h
24 h
Osmotic stress
Hormones etc
NaCl
Mannitol
20% PEG6000
Low Cl-
pH 4.5
100 µM AlCl3
Darkness
Light
H2O2
ABA
IAA
SA
GA3
MeJA
GABA
Increase > 8-fold
Increase < 8-fold
No change
Decrease to 12.5%
Decrease to less than 12.5%

Figure S3

Figure S3 Summary of *OsALMT4* expression in rice grown under various treatments.

OsALMT4 expression in the root and shoot tissue of 14 d old rice seedlings grown in hydroponics was monitored after 6 h and 24 h exposure to different abiotic treatments including 200 mM NaCl, 350 mM mannitol (same osmolarity as the NaCl treatment), 20% PEG6000, low Cl^-, continual light or darkness and H_2O_2. Another set of treatments included hormones such as abscisic acid (ABA), gibberellin (GA_3), indole acetic acid (IAA), salicylic acid (SA), methyl jasmonic acid (MeJA) (all 100 µM) and 1 mM γ amino butyric acid (GABA) added to the nutrient solution. Plants in control treatment were harvested at each time point for comparison. The heat map summarises the significant changes in expression between control and transgenic plants where dark blue is a >8-fold increase, light blue is an increase <8-fold, grey indicates no significant change, pink is a decrease down to 12.5% of control and red is a decrease to less than 12.5% of control.

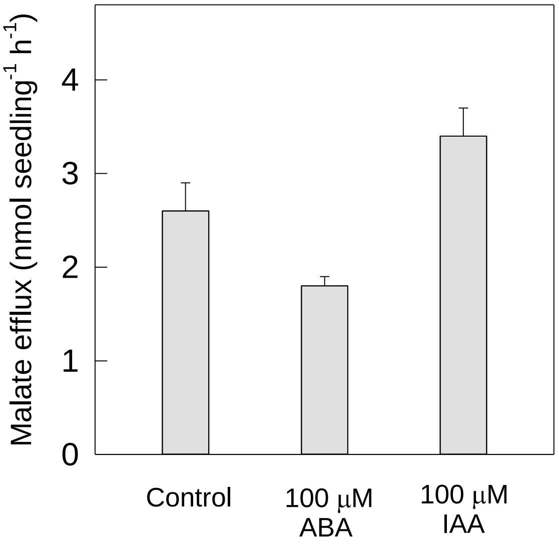

Figure S4 Effect of ABA and IAA on malate efflux from transgenic rice

Malate efflux from transgenic line OX5 over-expressing *OsALMT4* was measured without and with plants was measured after exposure to various treatments. OX5 seedlings over-expressing *OsALMT4* were grown in flasks with sterile nutrient solution (pH 5.6) and placed in a growth room with 50 µmol $m^{-2}s^{-1}$ light intensity. After four days the solution was replaced with 15 ml of control solution (0.5 mM $CaCl_2$, pH 5.6) or control solution with added 100 µM ABA or IAA. After 24 h malate concentrations in the flask were measured with an enzyme assay. Data are mean and SE (n = 3). No significant differences were detected between the treatments.

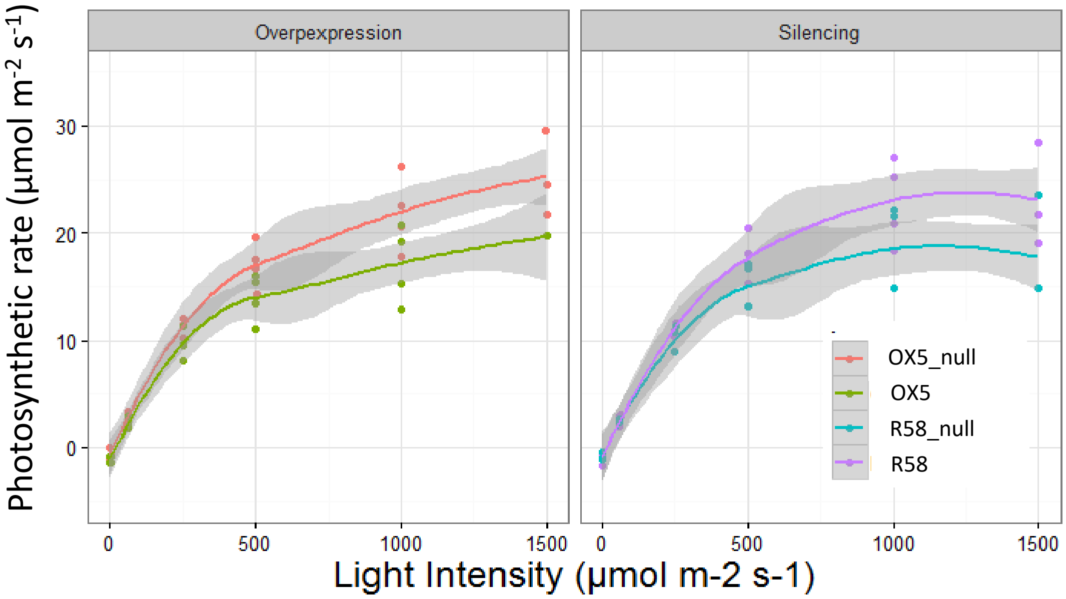

Figure S5 Photosynthetic responses to irradiance

The responses of CO_2 assimilation rates to varying irradiance were measured on fully expanded flag leaves of the transgenic lines, with either reduced (R58) or increased (OX5) *OsALMT4* expression and their relative null segregate plants (n = 4). Plants were grown under flooded soil condition for six weeks in a glasshouse with natural lighting and a 32/24°C temperature range. Photosynthetic measurements were performed as described in Materials and Methods. The individual points in the scatter plots represent assimilation rates of each individual leaf. The polynomial regression line was added with the fitting function "geom_smooth" of the R package "ggplot2". Shaded area represents level of confidence interval of 0.95.
