# Supplementary material for: Altered Expression of the Malate-Permeable Anion Channel OsALMT4 Reduces the Growth of Rice Under Low Radiance
Source: Front Plant Sci. 2018 May 3;9:542. doi: 10.3389/fpls.2018.00542 (PMC5943490; doi:10.3389/fpls.2018.00542)
Supplement: Supplementary file 2 [file Data_Sheet_2.pdf]

OsALMT4 expression affect growth in low light by Liu *et al*

SUPPLEMENTARY TABLES

Table S1 Biomass of transgenic rice lines after growth in various treatments.

OX and RNAi lines and their nulls were grown in hydroponics with 100 mM NaCl, 50 mM mannitol or a low-Cl^- solution and total biomass measured after 28 d. Each experiment has its own control as indicated. Light experiments, were performed in chambers with maximum diurnal light intensity of 700 µmol $m^{-2}s^{-1}$ or 300 µmol $m^{-2}s^{-1}$ in flooded pots. Shoot biomass was measured after 56 d for the OX lines and after 28 d for the RNAi lines. Data show mean and SE (n = 5-6).

**OX lines**				
	Control	***100 mM NaCl***	***Control***	***50mM Mannitol***
OX5	0.40 ± 0.09	0.12 ± 0.01	0.40 ± 0.09	0.18 ± 0.02
OX5_null	1.11 ± 0.18	0.40 ± 0.04	1.11 ± 0.18	0.65 ± 0.07
OX2	0.78 ± 0.04	0.10 ± 0.02	0.78 ± 0.04	0.36 ± 0.05
OX2_null	1.21 ± 0.17	0.35 ± 0.02	1.21 ± 0.17	0.69 ± 0.04
	Control	***Low Cl-***	***Control (High light)***	***Low light***
OX5	0.48 ± 0.06	0.33 ± 0.04	9.23 ± 0.73	4.93 ± 0.19
OX5_null	1.17 ± 0.1	0.63 ± 0.1	13.98 ± 2.19	11.62 ± 1.04
OX2	0.73 ± 0.05	0.50 ± 0.04	9.42 ± 0.69	2.75 ± 1.7
OX2 null	1.16 ± 0.07	0.68 ± 0.02	13.6 ± 0.92	9.92 ± 0.61
**RNAi lines**				
	Control	***100 mM NaCl***	***Control***	***50mM Mannitol***
R24	0.89 ± 0.04	0.52 ± 0.04	1.7 ± 0.11	1.17 ± 0.03
R24_null	0.83 ± 0.03	0.51 ± 0.04	1.44 ± 0.14	0.95 ± 0.16
R58	0.62 ± 0.03	0.39 ± 0.01	1.61 ± 0.15	1.03 ± 0.04
R58_null	0.87 ± 0.06	0.49 ± 0.04	1.83 ± 0.27	1.25 ± 0.14
	Control	***Low Cl-***	***Control (High light)***	***Low light***
R24	0.46 ± 0.07	0.26 ± 0.02	4.6 ± 0.24	0.33 ± 0.06
R24_null	0.54 ± 0.04	0.35 ± 0.02	4.72 ± 0.22	1.3 ± 0.16
R58	0.52 ± 0.01	0.24 ± 0.04	4.39 ± 0.16	0.2 ± 0.04
R58_null	0.38 ± 0.05	0.29 ± 0.03	4.23 ± 0.29	1.02 ± 0.08

Table S2 Metabolomic analysis of sugars in transgenic rice lines with altered levels of *OsALMT4* expression.

The concentrations of sugars in the shoots and leaves of hydroponically grown rice plants. OX5 is a homozygous transgenic line with increased *OsALMT4* expression and R24 is a homozygous transgenic line with reduced *OsALMT4* expression. OX5_null and R24_null are the null segregant lines. Data show the mean and SE (n = 4). No significant differences were detected between the transgenic and null plants.

Rice Lines	OX5	OX5_null	R24	R24_null
		(pmol mg^{-1} tissue)		
Shoots				
Erythritol	172 ± 17	140 ± 19	129 ± 15	116 ± 10
Xylose	103 ± 6	114 ± 11	115 ± 2	94 ± 6
Arabinose	245 ± 15	223 ± 27	282 ± 16	194 ± 16
Ribose	123 ± 5	113 ± 19	172 ± 12	131 ± 14
Arabitol	142 ± 6	142 ± 12	143 ± 24	131 ± 7
Fucose	558 ± 17	537 ± 30	519 ± 16	462 ± 32
Mannose	105 ± 5	96 ± 4	103 ± 8	95 ± 9
Mannitol	61 ± 3	443 ± 388	66 ± 7	58 ± 6
Glucuronate	75 ± 3	76 ± 7	79 ± 3	70 ± 5
Galactitol	154 ± 6	146 ± 21	165 ± 17	138 ± 8
Gluconate	548 ± 89	579 ± 76	520 ± 38	585 ± 94
Inositol	3360 ± 224	3126 ± 117	2611 ± 84	2785 ± 309
Maltose	279 ± 21	284 ± 26	323 ± 23	398 ± 51
Trehalose	255 ± 46	201 ± 27	233 ± 17	222 ± 33
β-Gentibiose	295 ± 11	290 ± 46	372 ± 8	266 ± 30
Melibiose	126 ± 7	117 ± 4	108 ± 3	104 ± 5
Raffinose	1042 ± 162	749 ± 74	421 ± 65	603 ± 121
Erlose	52 ± 5	46 ± 2	41 ± 1	43 ± 2
Melezitose	54 ± 3	46 ± 1	53 ± 3	44 ± 2
Fructose	36821 ± 1164	36245 ± 3424	37694 ± 3191	29199 ± 3829
Glucose	46282 ± 1368	44398 ± 3987	47006 ± 3092	38166 ± 4201
Inositol	4948 ± 463	4488 ± 230	3035 ± 146	3621 ± 569
Sucrose	55485 ± 559	50750 ± 2018	42726 ± 1251	45607 ± 3402

Roots				
Xylose	155 ± 11	227.16 ± 33	160 ± 27	191 ± 21
Arabinose	1550 ± 119	1782.07 ± 60	1811 ± 172	1695 ± 82
Ribose	325 ± 34	405.21 ± 41	340 ± 37	336 ± 27
Fucose	50 ± 3	53.82 ± 3	47 ± 1	53 ± 4
Mannose	192 ± 21	223.18 ± 18	148 ± 9	183 ± 31
Galactose	106 ± 8	119.24 ± 11	132 ± 18	122 ± 6
Glucuronate	68 ± 7	94.01 ± 11	56 ± 6	70 ± 13
Gluconate	194 ± 32	258.19 ± 18	272 ± 37	273 ± 26
Inositol	878 ± 93	1019.5 ± 79	790 ± 59	853 ± 154
Maltose	94 ± 17	113.85 ± 16	79 ± 9	58 ± 6
Trehalose	514 ± 44	542.82 ± 23	478 ± 18	502 ± 11
β-Gentibiose	258 ± 22	291.15 ± 29	301 ± 29	306 ± 29
Melibiose	250 ± 12	291.03 ± 17	243 ± 17	213 ± 15
Raffinose	154 ± 7	168.15 ± 16	111 ± 14	123 ± 8
Melezitose	71 ± 5	90.55 ± 3	74 ± 4	68 ± 1
Fructose	28282 ± 2542	39067.87 ± 6463	34863 ± 5203	34574 ± 6705
Glucose	28646 ± 983	30359.72 ± 2676	30482 ± 1905	30253 ± 1133
Sucrose	32080 ± 2103	36425.27 ± 1050	26617 ± 1423	23907 ± 1794
